# Experience with bioresorbable splints for treatment of airway collapse in a pediatric population

**DOI:** 10.1016/j.xjtc.2021.04.010

**Published:** 2021-04-19

**Authors:** Ali Kamran, Charles J. Smithers, Christopher W. Baird, Russell W. Jennings

**Affiliations:** a Department of General Surgery, Boston Children's Hospital, Harvard Medical School, Boston, Mass; b Department of General Surgery, Johns Hopkins All Children's Hospital, St Petersburg, Fla; c Department of Cardiac Surgery, Boston Children's Hospital, Harvard Medical School, Boston, Mass

**Keywords:** tracheobronchomalacia, airway collapse, external airway splinting, bioresorbable splint, CT, computed tomography

## Abstract

**Objective:**

To report our experience with novel external tracheal and bronchial placed bioresorbable splints in children with severe symptomatic airway collapse.

**Methods:**

Retrospective review of patients undergoing bioresorbable splint placement.

**Results:**

Between July 2018 and February 2020, 14 patients received 16 external splints (trachea, n = 8; left bronchus, n = 7; and right bronchus, n = 1). Preoperatively, 7 patients had a tracheostomy; 6 of them were receiving mechanical ventilation with ventilator settings so high that they required an inpatient setting, often in an intensive care unit. Median age at implant was 14.5 months (range, 2 months-14 years). Splints were formed from moldable bioresorbable plates (RapidSorb; Synthes, Oberdorf, Switzerland) and were customized intraoperatively around a Hegar dilator. A series of Prolene sutures were placed through into the airway cartilage under simultaneous bronchoscopic and direct visualization and then tied securing the airway within the splint. Concomitant procedures were also performed in the region of the airway splints, consisting of airway reconstruction, cardiovascular procedures, and/or esophageal rotation (related to posterior tracheopexy). Median follow-up was 20 months (interquartile range, 12-21 months). Four patients required no further intervention. Although not necessarily in the splinted region, 7 patients required additional procedures, including posterior tracheobronchopexy (n = 2), temporary tracheal stent placement (n = 1), tracheal resection with end-to-end anastomosis (n = 1), closure tracheostomy (n = 1), and tracheostomy placement (n = 2). One patient required splint replacement and in 1 patient, the splint was removed later. All patients (except 2 deaths from unrelated causes) were discharged home. Three patients required mechanical ventilation at lower settings that allowed home ventilation (1 of those only at night), and 4 patients required tracheostomy collar. Indications for tracheostomy included subglottic stenosis, vocal cord paralysis, pulmonary insufficiency, small airway malacia, and laryngomalacia.

**Conclusions:**

An external bioresorbable splint can provide temporary external support while allowing the age-proportional growth of the airway. We applied readily available bioresorbable plates that were custom-molded based on the location, shape, and length of the collapsing airway in selected patients presenting with severe tracheobronchomalacia from loss of structural support and/or cartilage deformation. Further study that includes long-term outcomes are necessary to define the best role for these external splints as part of comprehensive airway management.

**Figure:**
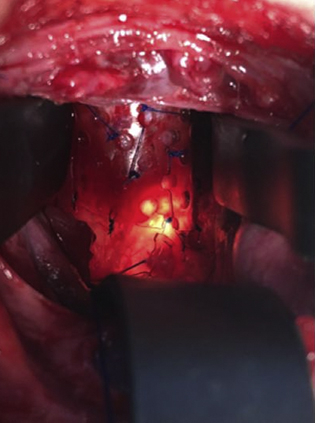
Bioresorbable custom-molded splint secured around a severely collapsed area of the trachea.

**Central Message**

External airway stabilization using a bioresorbable splint is a novel surgical approach to treat selected children with severe symptomatic airway collapse.

**Perspective**

External airway splinting can be an effective therapeutic strategy in selected patients with severe tracheobronchomalacia not amenable to other surgical options. External tracheal and bronchial splints made from readily available moldable bioresorbable plates can be applied in localized areas of the airway with life-threatening collapse.

See Commentary on page 170.

Airway obstruction in children can result from different etiologies, including congenital tracheal rings, tracheobronchomalacia, and/or vascular compression.^1^, ^2^, ^3^ The effects can lead to ineffective ventilation and reduced clearance of secretions, resulting in a broad spectrum of respiratory symptoms, including noisy breathing, chronic cough, exercise intolerance, feeding difficulties with shortness of breath, and prolonged pulmonary infections that can progress to bronchiectasis. In severe cases, patients may also present with episodes of severe respiratory distress and/or acute life-threatening events and may require intubation of the airway and high ventilator pressures to ensure adequate ventilation.

In patients with vascular compression syndrome and/or tracheobronchomalacia, surgical treatment has been reserved for symptomatic patients with bronchoscopic findings of severe airway collapse who have failed maximum medical therapy. Historically, aortopexy has been the primary surgical option whereby the airway compression is partly or completely relieved, but airway expansion is not always ensured.^4^^,^^5^ More recently, anterior and posterior tracheobronchopexy have been applied externally to directly open the airway^6^, ^7^, ^8^, ^9^, ^10^; however, a subset of children may still experience persistent airway collapse due to deformed cartilaginous tracheal or bronchial segments, particularly lateral compression deformities that cannot be corrected with anterior-posterior pexy procedures. As an alternative, external rigid prosthesis (eg, external stents, grafts, and splints), have been used to provide direct external airway stabilization without disrupting the membrane thus minimizing the risk of granulation. However, many of these external adjuncts are nonabsorbable and growth limiting. In this study, we report our experience with novel external tracheal and bronchial bioresorbable splints in children with severe symptomatic airway collapse.

## Methods

With institutional review board approval, a retrospective review was conducted of all patients with symptomatic airway collapse surgically treated with the placement of an external bioresorbable splint at Boston Children's Hospital and Johns Hopkins All Children's Hospital between July 2018 and February 2020 (IRB-P00004344). Data retrieved included patients' demographic characteristics, comorbidities, prior surgical history, preoperative respiratory clinical status, bronchoscopic evaluation before and after splint placement, surgical procedures performed, and postoperative outcomes.

Preoperative evaluation in all patients included 3-phase dynamic bronchoscopy to thoroughly assess the airway structure and the location and severity of the airway collapse,^1^^,^^11^ and computed tomography (CT) angiogram to identify great artery anomalies, delineate the anatomic relationships of the trachea and mainstem bronchi to the surrounding vasculature and spine, and to identify any other mass lesions near the airway.

The surgical plan was customized in each patient based on the location, character, and degree of airway collapse, considering the underlying pathologies, clinical concerns, and combined conditions, including cartilage deformation and malformation, vascular anomalies causing symptomatic airway compression (such as aberrant arteries, vascular rings, and circumflex aorta), and chest wall and/or spine deformities causing a significant decrease in anterior-posterior thoracic space. Frequently, the decision to apply the splint was made intraoperatively. Our preference was to correct all the airway lesions and other comorbidities in a comprehensive surgical approach to improve airway outcomes and prevent multiple reoperations.

Procedures performed to treat tracheobronchomalacia and/or airway compression included posterior tracheobronchopexy (to directly address posterior membranous intrusion), anterior tracheobronchopexy (to directly address anterior airway compression), anterior aortopexy (to relieve anterior airway compression by the aorta), posterior descending aortopexy (to relieve posterior left mainstem bronchus compression by the descending aorta located too far anteriorly from the spine), slide tracheobronchoplasty (to correct airway critical stenosis from complete cartilaginous rings), and placement of external bioresorbable splints (to support the tracheobronchial structure in patients with complex airway collapse not alleviated by other surgical procedures).

**Figure 1:**
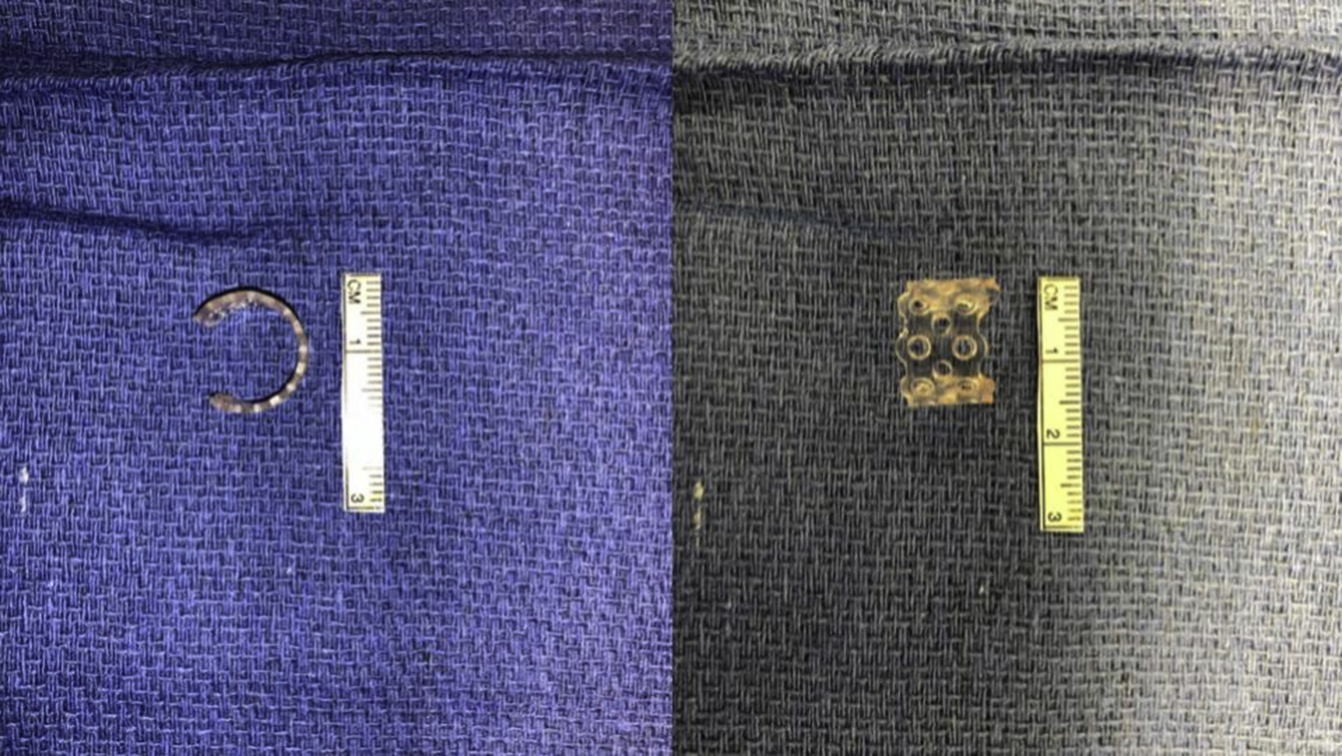
The bioresorbable plate was cut and molded intraoperatively according to the length and shape of the desired airway area.

**Figure 2:**
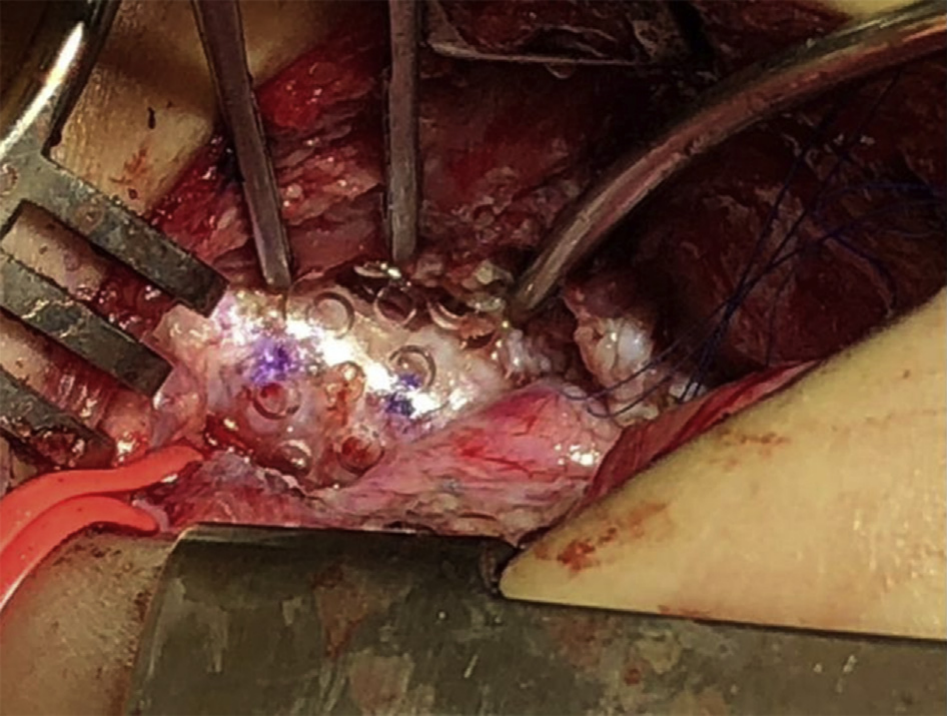
The bioresorbable custom-molded splint placed to fit best around the collapsing area.

Splints were formed from readily available moldable bioresorbable plates (RapidSorb; Synthes, Oberdorf, Switzerland) and were customized intraoperatively around a Hegar dilator 2 to 3 mm larger than the external diameter of the airway after dipping into 70°C saline solution (Figure 1). After shaping the splint to fit best around the collapsing area, a series of sutures were placed under simultaneous bronchoscopic and direct visualization. The sutures were then passed through the splint's interstices, and the splint was parachuted down to the airway. The sutures were then tied, securing the airway within the splint (Figure 2). Patients had a bronchoscopic evaluation after chest closure.

## Results

Between 2018 and 2020, 14 patients (9 male) who underwent surgical treatment at our centers for symptomatic airway collapse received external bioresorbable splint at a median age of 14.5 months (range, 2 months-14 years) (Table 1). All patients were symptomatic and had bronchoscopic evaluation demonstrating severe airway narrowing due to tracheobronchomalacia and/or airway compression. Seven patients had tracheostomies, 6 of them were dependent on mechanical ventilation with pressures too high to go home. Seven patients had cardiovascular comorbidity causing airway compression, and there were 2 patients with a significant decrease in thoracic space due to a chest wall deformity.

**Table 1:** Demographic characteristics

Patient no.	Age	Gender	Comorbidities	Prior operation(s)	Respiratory symptoms and clinical status
1	14 y	Female	DiGeorge syndrome, TOF with absent pulmonary valve, LPA stenosis, Pectus excavatum, LMB compression (by LPA stent), TBM	• TOF repair • LPA angioplasty • LPA stent placement	Exercise intolerance
2	2 mo	Male	Goldenhar syndrome, RPA agenesis (right lung hypoperfusion), LPA sling, Tracheal stenosis (complete tracheal rings), LMB, RMB compression (by LPA sling)	None	MV dependent (PIP, 20; PEEP, 9)
3	2 y	Female	CDH (Morgagni), TBM	• CDH repair	Multiple episodes of respiratory arrest, recurrent pulmonary infections
4	5 mo	Male	XYY syndrome, Laryngomalacia (2B), TBM	• Supraglottoplasty • Posterior tracheobronchopexy (T2, T3, RMB, LMB)	Feeding difficulty with shortness of breath and cough, noisy breathing, multiple episodes of desaturation
5	4 mo	Male	TOF with absent pulmonary valve, LPA stenosis, tracheal stenosis (complete tracheal rings), LMB compression (by LPA stent), LMB bronchomalacia	• TOF repair, LeCompte procedure, LPA replacement and stent placement, tracheal resection with flat tracheoplasty	MV dependent (PIP, 30; PEEP, 6)
6	5 mo	Male	EA/TEF type C (VACTERL syndrome), Laryngeal cleft (type 2), TBM	• EA/TEF repair (at OSH) • Posterior tracheobronchopexy (T2, T3, RMB, LMB), Posterior descending aortopexy, tracheal diverticulum resection, esophageal stricturoplasty	MV dependent (PIP, 15; PEEP, 5)
7	2 mo	Male	DiGeorge syndrome, TOF with absent pulmonary valve, right-sided aortic arch, LMB, RMB compression (by dilated PAs), TBM	None	MV dependent (PIP, 28; PEEP, 10)
8	3 y	Female	HLHS, coarctation of the aorta, LMB compression (by severely dilated ascending aorta and transverse arch)	• Biventricular reconstruction surgeries	
9	3 mo	Male	EA/TEF type C Laryngeal cleft (type 3) TBM	• EA/TEF repair (Foker procedure) • Laryngeal cleft repair, posterior tracheobronchopexy (T1, T2, T3, RMB, LMB), posterior descending aortopexy	MV dependent (PIP, 19; PEEP, 7)
10	8 mo	Female	EA/TEF type C (VACTERL syndrome) TBM	• EA/TEF repair (Foker procedure) • Posterior tracheobronchopexy (T1, T2, T3, RMB, LMB), tracheal diverticulum resection, division of acquired esophageal-pulmonary fistula, esophageal anastomotic stricture resection and end-to-end reanastomosis	MV dependent (PIP, 27; PEEP, 10)
11	3 y	Male	HLHS, LMB compression (by LPA stent)	• Biventricular reconstruction surgeries	
12	2 y	Female	Au-Kline syndrome, congenital heart disease (multiple VSDs), bronchopulmonary dysplasia, pulmonary HTN, decreased anterior-posterior thoracic area causing tracheal and LMB compression	• PA banding followed by multiple VSDs closure and band take-down with some residual VSDs	Oxygen dependent, recurrent pulmonary infections
13	2 y	Male	LMB compression from esophagus, TBM	None	Recurrent respiratory infections
14	2 y	Male	H-type TEF, aberrant right subclavian artery vocal cord paralysis TBM, subglottic stenosis	• TEF repair (2 times), tracheal diverticulum resection, • Division aberrant right subclavian artery • Posterior tracheopexy (T1, T2, T3)	Tracheostomy dependent

*TOF*, Tetralogy of Fallot; *LPA*, left pulmonary artery; *LMB*, left mainstem bronchus; *TBM*, tracheobronchomalacia; *RPA*, right pulmonary artery; *RMB*, right mainstem bronchus; *MV*, mechanical ventilation; *PIP*, peak inspiratory pressure; *PEEP*, positive end-expiratory pressure; *CDH*, congenital diaphragmatic hernia; *T1*, upper cervical trachea; *T2*, middle thoracic trachea; *T3*, distal thoracic trachea; *EA/TEF*, esophageal atresia/tracheoesophageal fistula; *VACTERL*, Vertebral Anorectal Cardiac Tracheo-esophageal Renal Limb anomaly association; *OSH*, outside hospital; *PA*, pulmonary artery; *HLHS*,hypoplastic left heart syndrome; *VSD*, ventricular septal defect; *HTN*, hypertension.

Bioresorbable splints were placed at the trachea (n = 8), left mainstem bronchus (n = 7), and/or right mainstem bronchus (n = 1). Two patients had splints placed in more than 1 location. Intraoperative bronchoscopy revealed a significant improvement in the airway narrowing of the areas where the splints were placed, and the open airway area went from 10% ± 10% to 80% ± 15% of normal. All patients had concomitant procedures in the region of the airway splints, consisting of airway reconstruction, cardiovascular procedures, and/or esophageal rotation (related to posterior tracheopexy). Concomitant procedures included posterior tracheobronchopexy (n = 7), anterior tracheobronchopexy (n = 5), anterior aortopexy (n = 5), and slide tracheobronchoplasty (n = 2), and rotation esophagoplasty (n = 7). Six patients with a cardiovascular anomaly causing airway compression and 2 patients with a chest wall deformity had combined procedures during the same surgery. The surgical approach and procedures performed in each patient are presented in Table 2.

**Table 2:** Intraoperative characteristics

Patient no.	Surgical approach	Concomitant procedures performed	Airway location splint placed	Bronchoscopy	
				Before splint placement (% open)	After splint placement (% open)
1	Sternotomy	External splint placement, Anterior tracheobronchopexy (T2, T3, RMB), LeCompte procedure, LPA stent removal, LPA angioplasty, Nuss procedure	LMB	0	80
2	Sternotomy	External splint placement, slide tracheoplasty, Carina reconstruction, LPA reimplantation	T3 down to neo-carina and LMB	10	70
3	Cervical	External splint placement, tracheoplasty (T1: posterior mass resection), posterior tracheopexy (T1, T2), rotational esophagoplasty	T1	0	90
4	Sternotomy	External splint placement, posterior tracheobronchopexy (T2, T3, LMB), anterior aortopexy and PA pexy, rotational esophagoplasty	T2, T3 - LMB	30 (trachea) - 30 (LMB)	80 (trachea) - 50 (LMB)
5	Sternotomy	External splint placement, LMB slide bronchoplasty, PAs stent removal, PAs resection and replacement, left upper lobectomy	LMB	10	80
6	Sternotomy	External splint placement, posterior tracheopexy (T1), anterior tracheopexy (T1), anterior aortopexy, rotational esophagoplasty	T1	0	100
7	Sternotomy	External splint placement, posterior tracheobronchopexy (T2, T3, RMB, LMB), anterior tracheopexy (T2, T3), rotational esophagoplasty, TOF repair, PAs resection and replacement	RMB - LMB	0 (RMB) - 0 (LMB)	50 (RMB) - 70 (LMB)
8	Sternotomy	External splint placement, ascending aorta and transverse arch reconstruction	LMB	10-20	70-80
9	Sternotomy	External splint placement, anterior tracheopexy (T1, T2), anterior aortopexy and innominate artery pexy	T2	0	100
10	Sternotomy	External splint placement, anterior tracheobronchopexy (T2, RMB, LMB), Anterior aortopexy	T3	5-10	100
11	Sternotomy	External splint placement, Fontan procedure	LMB	5-10	90-100
12	Sternotomy	External splint placement, posterior tracheopexy (T2, T3), anterior aortopexy, rotational esophagoplasty, sternum reconstruction (rib harvest)	T2, T3	0-10	90
13	Thoracotomy (right)	External splint placement, posterior tracheobronchopexy (T3, LMB), rotational esophagoplasty	LMB	0-10	80
14	Cervical	External splint placement, posterior tracheopexy (T1), rotation esophagoplasty	T1	20	75

*T1*, Upper cervical trachea; *T2*, middle thoracic trachea; *T3*, distal thoracic trachea; *RMB*, right mainstem bronchus; *LPA*, left pulmonary artery; *LMB*, left mainstem bronchus; *PA*, pulmonary artery; *TOF*, tetralogy of Fallot.

With a median follow-up of 20 months (interquartile range, 12-21 months), 4 patients demonstrated resolution of respiratory signs and symptoms without any further tracheo-bronchial procedure. Seven patients required additional procedures, including some away from the splint: posterior tracheobronchopexy (n = 2), tracheostomy closure (n = 1), and tracheostomy placement (n = 2), and some in the splinted region: temporary tracheal stent placement (n = 1), tracheal resection with end-to-end anastomosis (n = 1) (Table 3). One patient required removal of the tracheal splint (Case #2). This patient had undergone extensive tracheal reconstruction, including slide tracheoplasty for long-segment tracheal stenosis attributable to complete tracheal rings, carinal bronchoplasty for bronchial size discrepancies, and external splint placement for severe tracheobronchomalacia as well as compression of the pulmonary artery sling. He had an open trachea after slide tracheoplasty and splint placement, but the right lung needed to be removed due to hypoperfusion and ventilation-perfusion mismatching, and the slide portion of the trachea subsequently developed relative stenosis. Therefore, he required tracheal resection and end-to-end anastomosis with a tracheal endoluminal stent placed and then ultimately removed with tracheostomy placement. In 1 patient, the bronchial splint required replacement because it was too thin and fractured (Case #4). There have been 2 patients with patent airways who died due to congenital comorbidities. All surviving patients were safely discharged home, 3 patients on lower settings that allowed home ventilation, 1 of those only at night, and 4 on tracheostomy collar. Tracheostomy indications included persistent cervical tracheomalacia (n = 3), severe distal bronchomalacia (n = 1), tracheal stenosis after reconstruction (n = 1), laryngomalacia and vocal cord paralysis (n = 1), and subglottic stenosis (n = 2). Some patients had more than 1 indication.

**Table 3:** Postoperative and follow-up details

Patient no.	Hospital LOS (d)	In-hospital respiratory adverse events	Additional procedures performed	Follow-up (mo)	Status
1	8	_	Chest wall reconstruction and expansion (Nuss bar removed, Ravitch procedure)	20	Good, much improved exercise intolerance Bronchoscopy: splint resorbed with 90% LMB narrowing due to posterior compression by descending aorta
2∗	234	Right lung ventilation–perfusion mismatch (due to right lung underlying pathology and pulmonary artery agenesis), tracheal stenosis after reconstruction	Right pneumonectomy, external splint removal, tracheal resection with end-to-end anastomosis, tracheal internal stent placement (removed 1 wk later), tracheostomy	21	Good, on trach (home ventilation PIP, 32; PEEP, 14)
3	21	Desaturation spells while crying	Posterior tracheobronchopexy (T2, T3, LMB), tracheostomy (persistent cervical tracheomalacia; 5 mo after splint placement)	28	Good, on trach collar (home humidified oxygen at night)
4	136	Bronchial splint fracture, subglottic stenosis	Bronchial splint replacement, posterior bronchopexy (LMB, revision), tracheostomy	27	Good, on trach (home ventilation at night PIP, 20; PEEP, 8)
5∗	155	Died (at 5 d postoperatively with patent airways; cause = congenital heart disease)	–	–	Died
6∗	114	Persistent cervical tracheomalacia	Tracheostomy	21	Good, on trach collar (home humidified room air)
7∗	176	Severe distal small airways bronchomalacia	Tracheostomy	11	Good, on trach (home ventilation PIP, 24; PEEP, 8)
8	16	–	–	19	Good
9∗	244	Laryngomalacia (residual laryngeal cleft), vocal cord paralysis	Tracheostomy	20	Good, on trach collar (home humidified room air at night)
10∗	273	–	–	18	Good
11	11	–	–	12	Good
12	167	Died (at 3 mo postoperatively with patent airways; cause = multiple congenital comorbidities)	–	–	Died
13	5	–	–	4	Good
14∗	28	Multilevel airway narrowing: Paralyzed left vocal cord, subglottic stenosis, persistent cervical tracheomalacia	Tracheostomy replaced	7	Good, on trach collar (home)

*LOS*, Length of stay; *LMB*, left mainstem bronchus; *PIP*, peak inspiratory pressure; *PEEP*, positive end-expiratory pressure; *T2*, middle thoracic trachea; *T3*, distal thoracic trachea.

∗ Tracheostomy- or mechanical ventilation-dependent at baseline.

## Discussion

Tracheobronchomalacia is a condition of dynamic collapse of the airway during respiration and is typically characterized by an excessive intrusion of the posterior airway membrane. Although imprecise and misleading, the term tracheobronchomalacia is frequently used to describe the static airway collapse due to external compression by adjacent structures (typically by blood vessels) and/or anterior or lateral cartilage deformation. The combination of dynamic posterior intrusion and static anterior or lateral compression can lead to complete airway collapse during forced expiration and in the most severe cases at rest or with minimal expiratory effort. The use of bioresorbable splints can provide the short-term external structural support necessary for the airway to reform with the potential for continued growth. Tracheobronchomalacia, external airway compression, and/or tracheobronchial malformation can lead to airway collapse, sometimes life-threatening. In patients with tracheobronchomalacia, normal changes in airway caliber during breathing are accentuated, and dynamic airway collapse occurs during forced expiration and coughing due to increased intrathoracic pressure. Because of the close anatomical relationship between intrathoracic structures, airway compression can be caused by surrounding structures, typically the blood vessels and the esophagus and particularly in patients who lack normal cartilaginous stiffness so that the airway structure becomes softer and more susceptible to collapse. Abnormal position or anatomy of the blood vessels (such as seen in patients with aberrant arteries, vascular rings, or circumflex aorta)^2^^,^^3^^,^^12^ or decrease in the thoracic cavity (such as seen in patients with chest wall or sternal deformities) can also have profound effects on external airway compression. The airway collapse in children encompasses various congenital or acquired causes and ranges broadly in clinical presentation.

Clinical presentation, in combination with bronchoscopic findings, helps to determine the indications and timing for treatment. Weakness of the airway cartilage tends to become more rigid with continued growth and avoidance of steroids. Minor degrees of airway collapse may improve as the child grows, often believed to be by age 24 months, although this is controversial. However, deformed cartilages and/or a broad posterior airway membrane may lead to severe airway collapse that may not improve with natural airway growth and maturation, and in fact, may worsen with time. All children influenced by mild to severe airway collapse benefit from medical management, including airway clearance protocols and avoiding steroids, while awaiting structural stability and luminal enlargement. Children with milder symptoms and less severe degrees of airway collapse may symptomatically improve as their growth continues and/or with conservative treatment. More severe cases often present with prolonged or recurrent respiratory infections, recurrent pneumonia requiring hospitalization, inability to extubate following illness or procedures, and/or life-threatening events. The majority of these children have <25% opening of 1 or more airway regions. Those with recurrent pulmonary infections typically have complete collapse of 1 or more airway regions, causing impaired mucus clearance from the airway distal to that region. Currently, we believe that all patients should undergo maximum medical therapy before committing to other interventions; however, inadequate response to medical management necessitates a more aggressive approach. For those children considered candidates for surgical intervention and who have failed maximum medical therapy, all other associated conditions, including vascular anomalies, chest wall deformities, mediastinal lesions, or other airway pathologies, should also be considered. Our preference is to correct the airway lesions at the same operation as other comorbidities, if possible, to prevent multiple reoperations with their attendant increased risks. For example, left pulmonary artery sling with severe cartilage deformation of the distal trachea resulting in 10% open airway should have both the vascular and airway reconstruction simultaneously to avoid a second operation. Another common example is esophageal atresia with coexisting severe tracheomalacia; these problems can be surgically repaired simultaneously as well.^13^

Children with airway collapse may present with signs and symptoms that are typically neither sensitive nor specific, and bronchoscopic evaluation remains the gold standard for an accurate diagnosis in symptomatic patients with a high index of suspicion. Our team at the Esophageal and Airway Treatment Center at Boston Children's Hospital has found 3-phase dynamic bronchoscopy (rigid or flexible) to be of the best utility to thoroughly assess the airway structure and the location and severity of the airway collapse.^1^^,^^11^ The first phase occurs while the patient is shallow breathing, demonstrating the basic anatomy of the airway as well as the airway compression, cartilage malformations, tracheobronchial lesions, and secretion accumulation. The second phase is to induce coughing and Valsalva maneuvers while observing the airway again, revealing the maximum dynamic airway collapse and the secretion accumulation that gets displaced from the distal airways and comes into the larger airways. This dynamic phase is critical for identifying the region and severity of dynamic tracheobronchomalacia. The third phase is to distend the airways to 40 to 60 cm water after aspirating all secretions, revealing the structures and lesions that may not typically be seen, such as tracheoesophageal fistula, tracheal diverticulum, and aberrant bronchi. A flexible 3-phase dynamic bronchoscopy with a small scope can be very useful if there is a concern for small airway collapse, which is often found in premature infants and children with bronchopulmonary dysplasia.

Historically, there have been a number of procedures proposed for patients with severe airway disease, including tracheal support surgery, external airway stenting, and splinting. The associated techniques have used materials, including bone autografts or free autologous rib grafts, polyethylene terephthalate, silicone-plastic, and polypropylene monofilament mesh. In the 1960s, Herzog and colleagues^14^ described the use of a free autologous rib graft to externally stent the adult trachea. The rigid rib was anchored to normal tracheal cartilage on either side of the affected segment and immobilized anteriorly by attachment to the rib.^14^ Rainer and colleagues^15^ employed techniques to support tracheal collapse associated with chronic obstructive lung disease in 23 adults with terminal emphysema. A polyethylene terephthalate-reinforced silicone-plastic prosthesis was sutured to the widened membranous portion of the trachea after a series of imbricating sutures were placed to narrow it.^15^ In children in the 1980s, Johnston and colleagues^16^ reported external stenting of the malacic airway segment with rib grafts that significantly reduced airway resistance allowing for tracheostomy decannulation. Several groups reported on the early use of surgically implanted polypropylene monofilament mesh splints to support the collapsing airway in children. Filler and colleagues^17^ were the first to report the use of airway splints in children where polypropylene monofilament mesh shaped into the desired semirigid form by cementing silicone-plastic rings to its surface was fashioned to partially encircle the airway. Vinogrod and colleagues^18^ then reported excellent results using polypropylene monofilament mesh in 6 patients with a mean follow-up of 5.3 years. Although they concluded long-term relief without compromising airway growth, concerns remained about the use of a material that incites such an inflammatory fibrous reaction and its chronic longer-term effects.

Currently, multiple options for surgical intervention are available, including procedures for relieving external airway compression caused by blood vessels and the esophagus (ie, anterior aortopexy, posterior descending aortopexy, and rotational esophagoplasty) and for directly addressing posterior membranous intrusion and anterior airway collapse (ie, posterior and anterior tracheobronchopexy).^1^^,^^4^, ^5^, ^6^, ^7^, ^8^, ^9^, ^10^, ^11^, ^12^, ^13^^,^^19^, ^20^, ^21^ Our center has the unique approach of combining these techniques with advanced reconstructive surgeries in cases with the vascular abnormalities causing airway compression (such as aberrant arteries, vascular rings, and circumflex aorta)^12^^,^^21^^,^^22^ or in cases with chest wall deformities resulting in a significant decrease in thoracic space (such as pectus excavatum). However, a subgroup of children may be found to have a complex airway collapse due to an excessive structural weakness and/or deformation that is not adequately alleviated by anterior or posterior aortopexy and tracheobronchopexy, which only apply anterior and posterior forces. In airways with lateral compression or deformation, lateral forces must be applied and the external splint uniquely accomplishes that goal, as well as anterior and posterior support when necessary.

More recent external splinting techniques using autologous or synthetic materials have been described for stabilizing collapsible airways.^23^ The need for airway growth in infants and children limits the solutions to resorbable or dilatable possibilities. External splints made from bioresorbable material have been introduced to temporarily provide external airway support while allowing age-proportional growth of the airway with full resorption predicted to occur within 1 to 3 years. Zopf and colleagues^24^ at the University of Michigan reported 3-dimensional printed, patient-specific external bioresorbable splinting for the treatment of tracheobronchomalacia in critically ill children. Using the patient's CT scan, computer software, and laser-based 3-dimensional printing system, a polycaprolactone splint is custom designed and then secured around the affected area to maintain airway support. In a recent case series, 15 patients (median age, 8 months) received 29 splints to treat severe tracheobronchomalacia with significant improvement at a median follow-up of 8.5 months.^25^

Our experience with the treatment of complex airway collapse using external bioresorbable splints reported herein included a series of 14 patients (median age, 14.5 months) with loss of structural airway support or cartilage deformation. We applied readily available bioresorbable plates that were custom-molded based on the location, shape, and length of the collapsing airway region. The bioresorbable plates (made from L-lactide and glycolide copolymers) are available in various sizes and thicknesses and are designed to resorb after approximately 9 to 12 months. The optimal plate shape and size is determined using bendable templates. The selected plate is cut, dipped into the 70°C saline solution, and molded according to the shape of the desired area on site at the operative field providing significant flexibility and adaptability. This intraoperative customization is a critical advantage of this strategy.

Side-to-side compression or malformations (scabbard deformities) and circumferential collapse deformities require lateral or circumferential support best provided with an external splint, and the size and shape of the external splint may not be predictable based on preoperative CT scans or other imaging. Using this technique, the splints can be modified as needed to improve the airway. We pursue direct tracheopexy options for all of our patients as the first-line surgical intervention but find that certain anatomic variants (scabbard or lateral deformities, circumferential collapse) to require additional support from the external splints. The use of external splints is synergistic with other techniques, including anterior and posterior airway pexies and vascular work. None of these techniques are independently able to accomplish all the goals in all patients and all segments of the airway. Many patients require different techniques to optimize each segment of the airway. Simultaneous bronchoscopy is used for all cases during repair to guide our choices and help prove that the combination of techniques chosen was effective in optimizing airway opening and support. For example, after vascular reconstruction, we sometimes see severe cartilage malformation that causes a lateral or transverse airway narrowing that is not amenable to correction by anterior-posterior forces via pexy procedures to open the airway. In these cases, we have the option of using the airway splint to provide the transverse forces to open the airway and induce cartilage development in the open position.

This series included very complex patients and had several concomitant procedures, which reinforced the utility of the intraoperative customizable bioresorbable splint to support the airways, often as the only viable option. We have not reoperated on any patients who were well, so we cannot objectively witness the dissolution of the splints, but we have no reason to doubt that the splints are resorbing as predicted, and the cartilaginous airways appear to be remodeling in a more open structure. The reintervention rate reflects our learning curve: One time we used too thin a splint and it fractured (replaced with thicker one) and also learning to use a splint 2 to 3 mm larger in diameter than the external diameter of the airway to provide support without compression. We also learned in the cervical splint cases that sometimes the mucosal flaccidity or redundancy can be as severe a problem as the cartilaginous collapse, and needs to be controlled as well. The scar tissue around the splints on re-exploration was apparent but not excessive. The vocal cord dysfunction in one patient (Case #9) highlights the need for intraoperative recurrent laryngeal nerve monitoring in both cervical and thoracic/mediastinal cases.

The results in this very complex group are good, and yet reflect the complexity and comorbidities that patients with anatomic airway anomalies often have. Although 7 patients had tracheostomies before and after surgery, the postoperative group was markedly improved. All patients (excluding 2 deaths from unrelated causes) were safely discharged home, 3 patients on lower settings that allowed home ventilation, 1 of those only at night, and 4 on tracheostomy collar. This is in contrast to the 7 patients preoperatively who had tracheostomies, 6 with ventilator settings so high that they required an inpatient setting, often in an intensive care unit. Indications for postoperative tracheostomy included subglottic stenosis, vocal cord paralysis, pulmonary insufficiency, small airway malacia, and laryngomalacia—all problems that are difficult to diagnose in the face of high airway pressures and mechanical ventilation, and not corrected by large airway support. Tracheostomy dependence in this group of patients should be temporary. These results reflect an early learning curve, and we believe support ongoing gathering and reporting of experience using this strategy. This approach may provide a potential solution for some very challenging problems faced by ear-nose-throat, cardiac, and thoracic surgeons facing complex airway compression or deformities.

## Conclusions

In the pediatric population with severe airway compression and/or deformation that cannot be corrected by anterior and/or posterior tracheobronchopexy, the use of external airway support may prove beneficial. Because the airway is expected to grow considerably with the child's growth, use of permanent external supports may lead to airway stenosis over time. For these reasons, we believe use of bioresorbable plates that can be molded and customized in the operative field provide a potential solution to allow airway growth and support. Given the wide variety of complex patients and airway anomalies, this approach should be utilized in centers with multidisciplinary expertise in airway reconstruction with close follow-up.

### Conflict of Interest Statement

The authors reported no conflicts of interest.

The *Journal* policy requires editors and reviewers to disclose conflicts of interest and to decline handling or reviewing manuscripts for which they may have a conflict of interest. The editors and reviewers of this article have no conflicts of interest.
